# Validation of electrochemiluminescent (ECL)-based immunoassay to determine the levels of antibodies against *Escherichia coli* O-lipopolysaccharide antigens

**DOI:** 10.1128/spectrum.00863-26

**Published:** 2026-09-08

**Authors:** Maanan Kamdar, Vichiksha Shah, Sarah Janssen, Elizabeth Garcia Dominguez, Todd Davies, Germie van den Dobbelsteen

**Affiliations:** 1 Bacterial Vaccines Discovery and Early Development, Johnson & Johnson Innovative Medicine 6808 , Raritan, New Jersey, USA; 2 Discovery and Manufacturing Statistics, Johnson & Johnson Innovative Medicine, Leiden, the Netherlands; 3 Bacterial Vaccines Discovery and Early Development, Johnson & Johnson Innovative Medicine, Leiden, the Netherlands; Emory University School of Medicine, Atlanta, Georgia, USA

**Keywords:** ExPEC, ECL validation, O-antigen, serum

## Abstract

Extra-intestinal pathogenic *Escherichia coli* (ExPEC) is a significant cause of invasive infections, including urinary tract infections, neonatal meningitis, and bacteremia. The emergence of multidrug-resistant ExPEC strains has intensified the need for effective prophylactic interventions. Johnson & Johnson has developed ExPEC10V, a multivalent vaccine targeting ten distinct O-antigens associated with pathogenic ExPEC strains. To assess the humoral immune response elicited by ExPEC10V, an initial enzyme-linked immunosorbent assay (ELISA) targeting *E. coli* O-polysaccharide (O-LPS) antigens was employed. However, given the vaccine’s multivalent antigenic composition, and to improve throughput and efficiency, a multiplex electrochemiluminescent (ECL) immunoassay was developed, qualified, and validated to enable simultaneous determination of IgG antibody titers against all 10 O-antigens, and the carrier protein (EPA) in human serum. The ECL assay was validated for relative accuracy, precision, and linearity using serum samples from an ongoing clinical trial. Precision of incurred samples was systematically assessed across multiple runs to ensure reliable detection of baseline antibody levels. Specificity was validated through competitive inhibition using homologous and heterologous O-LPS antigens. Assay performance was further confirmed by assessing total error, linearity, and dilutional linearity across a broad concentration range. Robustness was tested by varying critical incubation times, while ruggedness was demonstrated using two different ECL plate readers. Sample stability was verified under multiple freeze/thaw cycles and prolonged storage at 4–8°C. The assay demonstrated acceptable precision, relative accuracy, linearity, and robustness across all tested antigens. While most antigens met ruggedness and specificity criteria, O6A failed during simultaneous heterologous competition; however, acceptable specificity was achieved when tested with individual heterologous antigens. To further investigate the O6A outcome, various antigen coating concentrations were evaluated; no improvement in performance was observed at either higher or lower concentrations. Sample handling and storage conditions were comparable to reference conditions, and pre-coated plates remained stable for up to 3 years. *V*alidation of the ECL-based immunoassay for determining IgG antibodies titers against *E. coli* O-antigens and the EPA protein in human serum was successfully completed, with all parameters meeting predefined acceptance criteria. The assay offers a robust, sensitive, and efficient platform for evaluating vaccine-induced immune responses, supporting both clinical development and future research applications.

**IMPORTANCE:**

Extraintestinal pathogenic *E. coli* (ExPEC) is a major cause of serious infections, and growing antibiotic resistance makes these illnesses harder to treat. Vaccines such as ExPEC10V could help prevent these infections before they start, reducing the need for antibiotics and improving patient outcomes. To understand how well this vaccine stimulates protective antibodies, scientists need reliable tools to measure immune responses. Traditional tests could only evaluate one bacterial target at a time, which was slow and labor‑intensive for a vaccine containing 10 components. By developing a new multiplex electrochemiluminescent assay, we can now measure antibodies to all 10 ExPEC targets simultaneously using a very small amount of sample. This improvement increases efficiency, supports large clinical studies, and accelerates progress toward a preventive vaccine for an urgent global health need.

## INTRODUCTION

Extraintestinal pathogenic *Escherichia coli* (ExPEC) are gram-negative bacteria responsible for infections beyond the gut, including urinary tract infections (UTIs), neonatal meningitis, and septicemia (1, 2). ExPEC can lead to invasive *E. coli* disease (IED), which may progress to sepsis, septic shock, or death if untreated (3–6). Older adults (≥60 years) are at increased risk, with infections often being community-, hospital-, or healthcare-associated (7–13). A history of UTI caused by *E. coli* in 75–95% of cases further elevates IED risk (14, 15). The growing antimicrobial resistance among ExPEC strains has increased both the incidence and economic burden of IED (16, 17), which carries a case fatality rate of 12–18% (4, 11).

Surveillance in the USA and Europe identified 10 O-serotypes—O1A, O2, O4, O6A, O8, O15, O16, O18A, O25B, and O75—as responsible for ~65% of *E. coli* bloodstream infections (18). To address this, the ExPEC10V vaccine was developed, targeting these 10 surface-exposed O-antigen polysaccharides of *E. coli*, which are part of the outer membrane lipopolysaccharide and elicit opsonophagocytic antibody responses. The O-antigens are individually bioconjugated to a detoxified form of exotoxin A derived from *Pseudomonas aeruginosa* (EPA) as carrier protein. This vaccine was tested in a phase 1/2a study (NCT03819049) and was shown to be safe and immunogenic in older adults (19, 20).

The primary assay for evaluating vaccine-induced antibody responses was an ELISA targeting *E. coli* O-LPS. To improve throughput and efficiency, a multiplexed electrochemiluminescent (ECL) assay was developed. The ExPEC ECL immunoassay is designed in a multiplex format to allow the simultaneous determination of the levels of antibodies (total IgG) specific against O1A, O2, O4, O6A, O8, O15, O16, O18A, O25B, and O75 O-antigens, as well as the carrier protein EPA, in human serum samples. The assay combines high-binding carbon electrodes in a multi-spot microplate coated with the 11 different antigens. The antigen-specific antibodies present in serum samples are detected using a secondary antibody (anti-human IgG) labeled with SULFO-TAG. The application of electricity to the plate electrodes leads to light emission by the SULFO-TAG labels, and the light intensity measured is proportional to the levels of antigen-specific antibodies present in the serum samples. This assay enables simultaneous detection of multiple antigens, an advantage over traditional single-plex ELISA. Compared with ELISA and other multiplex platforms, the ECL assay offers an expanded dynamic range, improved sensitivity at low analyte concentrations, and reduced background interference in complex biological matrices. Additionally, ECL enables accurate single-dilution measurements, thereby conserving sample volume and facilitating higher analytical throughput. The ECL assay was organized into two panels: panel A included EPA, O1A, O2, O6A, and O25B; panel B included O4, O8, O15, O16, O18A, and O75. The assay was developed and qualified to measure antibodies against the 10 *E. coli* O-antigens and was used in a phase 1/2a study (NCT03819049) to assess ExPEC10V-induced antibody responses across all vaccine serotypes post-vaccination (19).

This article describes the subsequent validation of a multiplexed electrochemiluminescent (ECL) immunoassay for determining antibody titers against 10 *E. coli* O-antigens.

## MATERIALS AND METHODS

### Antigen preparation, coating optimization, and plate coating

For the ECL assay validation described in this manuscript, antigens consisted of purified lipopolysaccharides (LPS) derived from *Escherichia coli* serotypes O1A, O2, O4, O6A, O8, O15, O16, O18A, O25B, and O75. The antigens were supplied as lyophilized powders and were produced from inactivated in-house biomass using a single-phase extraction and purification process conducted by LPS-BioSciences (Orsay, France). Product quality was verified by NMR, MALDI-TOF MS, and HPLC. Prior to use, antigens were reconstituted in pyrogen-free water to a concentration of 5 mg/mL. The antigens were subsequently shipped to MSD (Rockville, Maryland), where multiplex plates were coated at a concentration of 20 ng/spot for each antigen, and coating optimization was performed to ensure intra- and inter-plate consistency. For each coating batch, three plates were sampled from the beginning, middle, and end of the production run. Human reference serum and blended antigen-specific antibodies were evaluated using a head-to-head comparison of conjugated and unconjugated formats to determine the final assay configuration. Assay performance was assessed by evaluating intra- and inter-plate coefficients of variation (CVs).

The ECL assay validation included the evaluation of multiple parameters to ensure the assay’s performance and reliability. Serum samples from study participants from the NCT03819049 trial were used for this validation study. As a core immunogenicity readout for the ExPEC10V candidate vaccine, the assay was validated in accordance with current regulatory guidelines and relevant industry white papers (21–23). The parameters tested were linearity, precision (including repeatability, intermediate precision, and ruggedness), precision of incurred samples, relative accuracy and total error, dilutional linearity, and the working range of the assay, i.e., the lower limit of quantification (LLOQ) and upper limit of quantification (ULOQ). Additional parameters assessed included specificity, robustness, sample stability, plate stability, and precision of low-concentration unimmunized sera.

The individual validation components are outlined in the following sections.

#### Precision, linearity, and relative accuracy

To validate assay linearity, precision, and relative accuracy/total error, three pools of high-titer serum samples from subjects vaccinated with ExPEC10V were tested. Each pool contained antibodies across all serotypes at varying concentration levels (high, medium, and low). For each pool, 10 different concentration levels were prepared using assay buffer, resulting in a total of 30 linearity samples per antigen. As recommended by the vendor (MSD), linearity was evaluated using assay buffer (MSD Diluent 100), which was selected as a commercially available alternative to IgG-depleted serum. In addition to the validation runs, a comparative assessment was performed using IgG-depleted serum as the diluent.

Precision was assessed using 12 incurred serum samples under conditions representative of routine testing. Repeatability and intermediate precision were calculated as the coefficient of variation (%CV) using incurred samples, linearity samples, and high- and low-quality control samples to ensure consistent performance across the assay range.

Ruggedness was evaluated as part of the precision assessment by incorporating an additional source of variability, namely the use of two different ECL plate readers. Acceptance criteria for ruggedness were defined as ≤30% CV for both repeatability and intermediate precision at the individual sample and overall levels.

Relative accuracy was determined by comparing measured values to expected concentrations based on the linearity samples. Additionally, for each linearity sample, the total error (%TE) was calculated as the sum of the %CV for IP and the absolute value of the relative error (%RE). The %TE should not exceed 50% for samples with concentrations within the assay range.

#### Determination of the analytical measurement range

The LLOQ and ULOQ were defined as the lowest and highest concentrations within the linear range that meet criteria for relative accuracy and precision: (i) the linear range is where the 90% confidence interval (CI) of the slope falls between 0.80 and 1.25. (ii) Acceptable precision (%CV IP ≤30%) and total error (%TE ≤50%) were used as acceptance criteria.

With the analytical range determined, dilutional behavior was then examined to support the measurement of higher-titer samples.

#### Dilutional linearity assessment

Dilutional linearity was assessed to determine the maximum allowable pre-dilution factor for clinical samples with high antibody concentrations. Samples were diluted at factors of 1,000×, 5,000×, and 25,000× and tested in duplicates across six independent runs by three operators using two distinct plate layouts. This design yielded 12 reportable values per dilution level and sample. Given the defined assay range, it was necessary to demonstrate that samples with concentrations exceeding the ULOQ could be accurately quantified after dilution. A dilution factor was considered acceptable if it fell within the dilutional linear range and the intermediate precision (%CV IP) was ≤30%. Next, the selectivity of the assay was determined through antigen competition experiments.

#### Specificity assessment

Specificity was evaluated using eight incurred samples per assay via competitive inhibition with homologous and heterologous O-LPS antigens representing the 10 O-antigens and the EPA carrier protein. The competition was performed with 200 ng/antigen/well, corresponding to a 10-fold increase from the current coating concentration used in the assays for all antigens (20 ng/mL). Three assessments were performed: (i) Homologous competition, measuring fold inhibition with the corresponding antigen; (ii) heterologous (pooled), measuring fold inhibition with all non-matching antigens simultaneously; and (iii) heterologous (individual), measuring fold inhibition with each non-matching antigen separately. Fold inhibition was calculated as the ratio of the uncompeted concentration to the competed concentration, i.e., Fold Inhibition = (Uncompeted concentration / Competed concentration).

#### Robustness assessment

Robustness was validated by varying incubation times for both serum samples and detection antibodies. A response surface design was employed, incorporating two continuous factors, serum incubation time and detection antibody incubation time, to assess main effects, two-way interactions, and quadratic terms. The design space was calculated where the assay is considered robust. This approach ensured that the assay’s performance remained consistent under minor procedural deviations.

#### Sample stability

Clinical sample stability was assessed under conditions representative of routine handling during collection, processing, and testing. In typical workflows, samples undergo a single thaw cycle followed by short-term storage at 4–8°C (~15 h); however, retesting may require additional freeze–thaw cycles or extended storage at 4–8°C or –80°C. Short-term stability was evaluated by comparing a reference condition (single freeze/thaw cycle) with stress conditions, including three and five freeze/thaw cycles, 72 h and 7 days of storage at 4–8°C, and combinations thereof.

Long-term stability was assessed over a period of up to 17 months by comparing antibody levels at 6, 12, and 17 months to baseline (0 months).

For each O-antigen serotype and the EPA protein, the mean difference and corresponding 90% confidence intervals were calculated to determine whether storage over time impacted assay performance or antibody detectability.

#### Plate stability

This assessment aimed to determine the maximum stability of pre-coated assay plates used to measure antibody binding responses to specific coated antigens. Plates were stored under manufacturer-recommended conditions and tested at multiple time points: day 0 (reference, corresponding to the initial plate batch) up to 3 years. At each time point, four plates were validated in separate assay runs to minimize potential bias from run-to-run variability. For each plate, six distinct serum samples and two quality control samples (high and low) were tested, resulting in a total of 32 measurements per time point across four independent runs.

## RESULTS

The validation outcomes for the key analytical performance characteristics are presented below. All parameters tested passed the target acceptance criteria, which were defined based on the current FDA guidelines and white paper available for these kinds of assays (23)

### Precision, linearity, and relative accuracy

The LLOQ and ULOQ for all antigens (EPA, O15, O16, O18A, O1A, O2, O25B, O4, O75, and O8) were established within the assay’s linear range, defined by acceptable precision (%CV IP ≤ 30%) and total error (%TE ≤50%) (Fig. 1).

**Fig 1:**
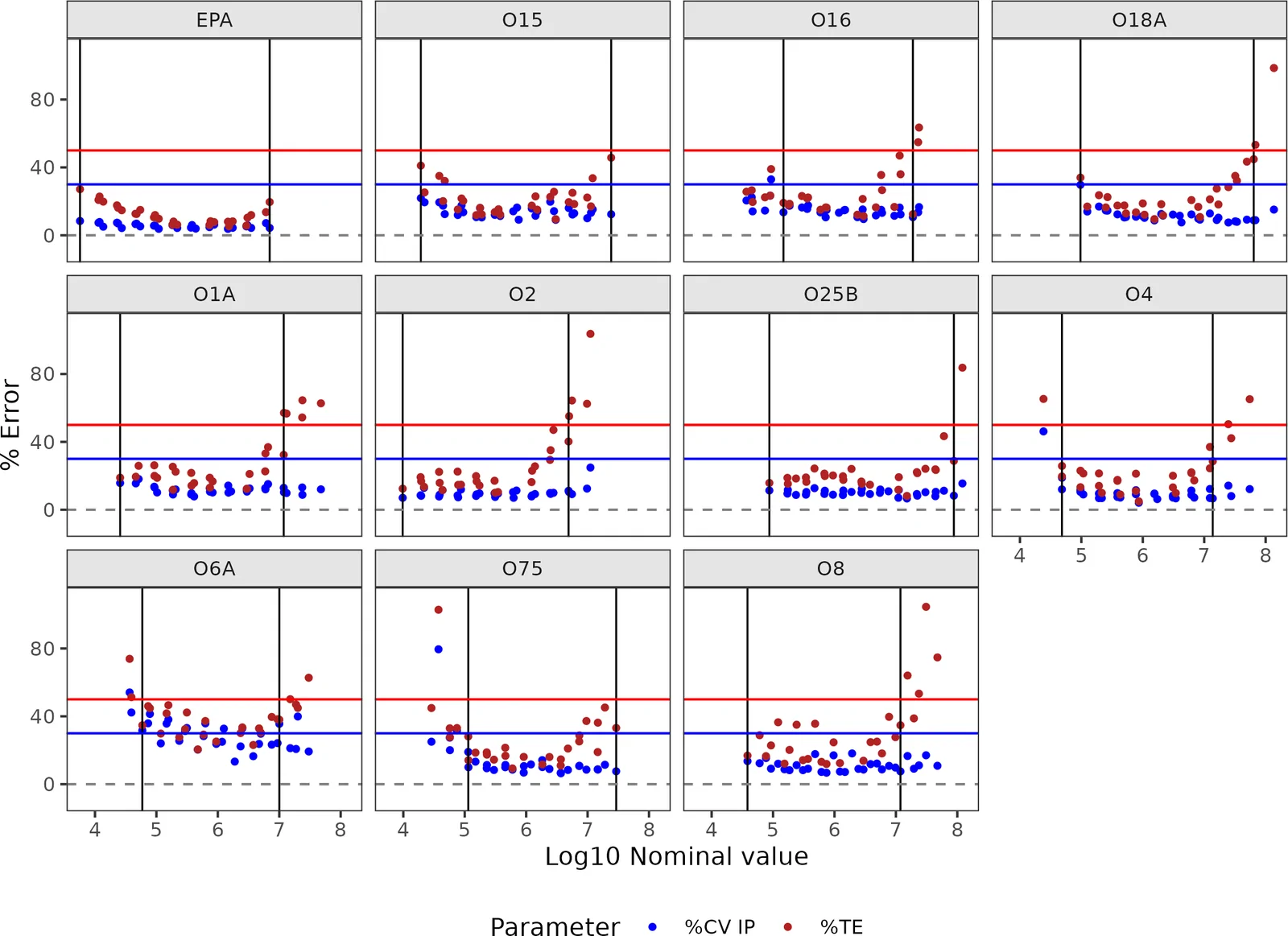
Graphical visualization of assay range based on %CV IP and %TE for all antigens included in the assay. The graph shows % error (Y-axis) versus log_10_ nominal value (X-axis). The blue and red values indicate %CV IP and %TE, respectively. The blue and red solid horizontal lines refer to the acceptance criteria equal to 30% (%CV IP) and 50% (%TE), respectively. The gray dashed horizontal line refers to % error equal to zero. The black solid vertical lines refer to the LLOQ and ULOQ, determined as the lowest and highest concentration level, respectively, within the linear range with %CV repeatability ≤30%, %CV IP ≤30% and %TE ≤50% for antigens EPA, O15, O16, O18A, O1A, O2, O25B, O4, O75, and O8, and with %TE ≤50% for antigen O6A.

Antigen O6A did not meet the precision criteria within the assay range (LLOQ–ULOQ). %CV repeatability and %CV IP values >30% were observed for samples with concentration levels within the assay range. The decision was made to set the assay range for O6A based on linearity (90% CI of the slope within 0.80 and 1.25) and relative accuracy (%TE ≤50%). For the remaining antigens, the assay ranges were set based on linearity, precision, and relative accuracy.

The overall precision evaluation, based on linearity and QC samples with a concentration level within the assay range, is described in Table 1. The overall %CV repeatability and %CV IP were estimated below 30% for all antigens including O6A. The overall %CV IP for O6A was estimated to be 28.60% as shown in Table 1.

**TABLE 1:** Overall assay precision^*a*^^,^^*b*^

Antigen	*N*	%CV repeatability	%CV between run	%CV IP	Pass
EPA	648	3.73	5.81	6.91	Yes
O15	648	12.08	8.88	15.04	Yes
O16	504	13.47	7.62	15.51	Yes
O18A	612	11.04	5.12	12.19	Yes
O1A	558	12.40	8.48	15.05	Yes
O2	576	8.22	6.52	10.51	Yes
O25B	630	8.88	7.73	11.79	Yes
O4	576	8.50	7.67	11.47	Yes
O6A	540	26.37	10.71	28.60	Yes
O75	558	9.16	7.95	12.15	Yes
O8	558	8.04	8.90	12.01	Yes

^*a*^ The variable “Pass” equals Yes when %CV repeatability ≤30% and %CV IP ≤30%. The variable “*N*” refers to the total number of available measurements.

^*b*^ The %CV repeatability, %CV between run, and %CV IP evaluation include linearity and QC samples with a concentration level within the assay range.

### Linearity assessment

All antigens exhibited acceptable linearity within the assay range, with the 90% confidence intervals for the slopes of the log_10_–log_10_ plots falling within the range of 0.80 to 1.25 (see Table 2). Only samples with a concentration level within the assay range are included in the linearity evaluation.

**TABLE 2:** Assay linearity evaluation

Antigen	*N*	Slope	Lower 90% CL	Upper 90% CL	Lower acceptance limit	Upper acceptance limit	Pass
EPA	540	1.03	1.03	1.04	0.80	1.25	Yes
O15	540	0.99	0.99	1.00	0.80	1.25	Yes
O16	396	1.04	1.03	1.05	0.80	1.25	Yes
O18A	504	1.04	1.04	1.05	0.80	1.25	Yes
O1A	450	1.04	1.04	1.05	0.80	1.25	Yes
O2	468	1.06	1.05	1.06	0.80	1.25	Yes
O25B	522	1.05	1.04	1.05	0.80	1.25	Yes
O4	468	1.04	1.03	1.04	0.80	1.25	Yes
O6A	432	1.02	1.01	1.04	0.80	1.25	Yes
O75	450	1.07	1.06	1.07	0.80	1.25	Yes
O8	450	1.06	1.06	1.07	0.80	1.25	Yes

### Analytical measurement range

Assay ranges were established based on precision and relative accuracy for all serotypes as shown in Table 3.

**TABLE 3:** Assay ranges

Serotype	LLOQ	ULOQ	Log_10_ LLOQ	Log_10_ LLOQ
EPA	5,704	6,981,511	3.76	6.84
O15	19,293	24,209,402	4.29	7.38
O16	147,727	18,909,043	5.17	7.28
O18	96,686	63,967,578	4.99	7.81
O1A	25,670	11,819,409	4.41	7.07
O2	9,771	4,896,620	3.99	6.69
O25B	87,114	87,820,768	4.94	7.94
O4	48,273	13,766,009	4.68	7.14
O6A	58,981	10,023,265	4.77	7.00
O75	114,175	29,228,865	5.06	7.47
O8	38,366	11,856,670	4.58	7.07

### Dilutional linearity assessment

Dilutional linearity was validated at dilution factors of 1,000×, 5,000×, and 25,000×. The 90% confidence intervals for the slopes of the dilutional linearity samples fell within the acceptable range of 0.80 to 1.25 for all antigens. A dilution factor was considered acceptable if it fell within the dilutional linearity range (90% CI of the slope within 0.80 to 1.25) and had a %CV IP ≤30% based on dilutional linearity samples with concentration levels within the assay range. Acceptable dilutions per antigen are listed in Table 4.

**TABLE 4:** Overview of acceptable dilution for retesting of clinical samples^*a*^

Antigen	Acceptable dilutions	Comment
EPA	No acceptable dilutions	
O15	1,000, 5,000, 25,000	
O16	1,000, 5,000, 25,000	
O18A	5,000 and 25,000	1,000 was non-evaluable
O1A	25,000	1,000 was non-evaluable
O2	1,000, 5,000, 25,000	
O25B	5,000 and 25,000	1,000 was non-evaluable
O4	5,000 and 25,000	1,000 was non-evaluable
O6A	5,000 and 25,000	1,000 was non-evaluable
O75	5,000 and 25,000	1,000 was non-evaluable
O8	5,000 and 25,000	1,000 was non-evaluable

^*a*^ In parallel with dilution performance, quality control behavior was assessed to support routine assay monitoring.

Although certain dilutions (e.g., 1,000× for some antigens) were below the assay’s measurable range and were therefore considered non-evaluable, some samples exhibited a %CV for intermediate precision (IP) greater than 30%, which is outside the acceptable range for precision.

### Quality control sample performance

High- and low-QC (HQC and LQC) samples were validated. Acceptance criteria were calculated to assess the validity of future assay runs based on 99.5% β-expectation tolerance intervals for the 2-out-of-2 rule (both high- and low-quality samples should pass the acceptance criteria), targeting a 99% probability of accepting a valid run. The %CV IP values for the HQC and LQC were within predefined limits (≤30%) for all serotypes.

The analytical findings were complemented by specificity testing to confirm discrimination at the antigen level.

### Specificity assessment

Specificity was validated through homologous and heterologous competition experiments (Fig. 2 and 3). Acceptance criteria required that at least 50% of samples exhibit a fold inhibition of ≥7 under homologous conditions and ≤3 under heterologous conditions. These criteria were met for the following antigens: EPA, O15, O16, O18A, O1A, O2, O25B, O4, O75, and O8. For O6A, only 25% of samples met the heterologous criterion when a mixture of competitor antigens was tested simultaneously. However, when heterologous antigens were assessed individually, O6A met the predefined acceptance criteria.

**Fig 2:**
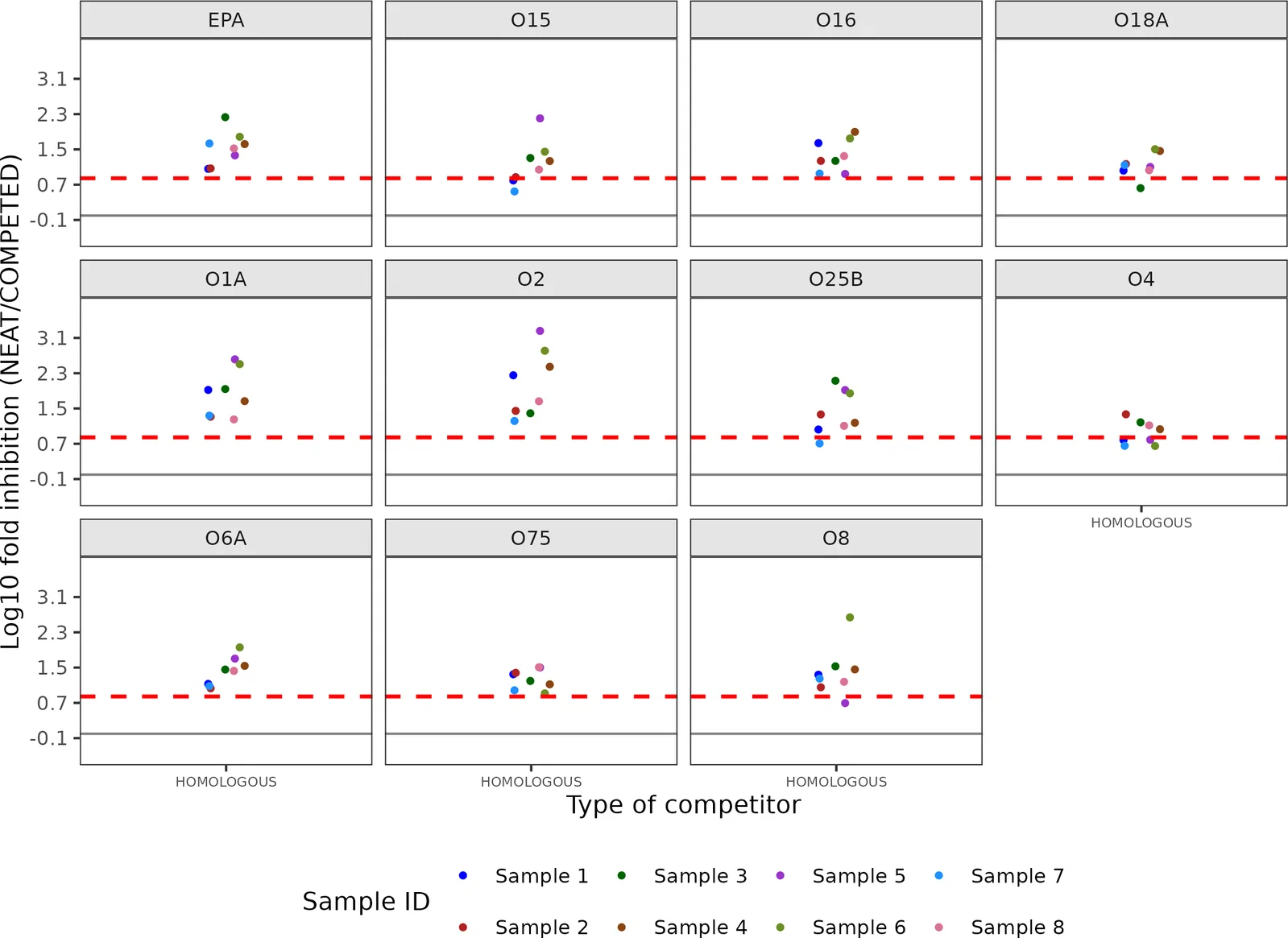
Evaluation of assay specificity with homologous antigen competition. Log_10_ fold inhibition (neat/competed), Y-axis for homologous competition. The colors indicate the sample ID. The gray horizontal solid line refers to a log_10_ fold inhibition equal to zero, and the red horizontal dashed line refers to the log_10_ acceptance criteria for homologous competition, i.e., log_10_ 7-fold inhibition.

**Fig 3:**
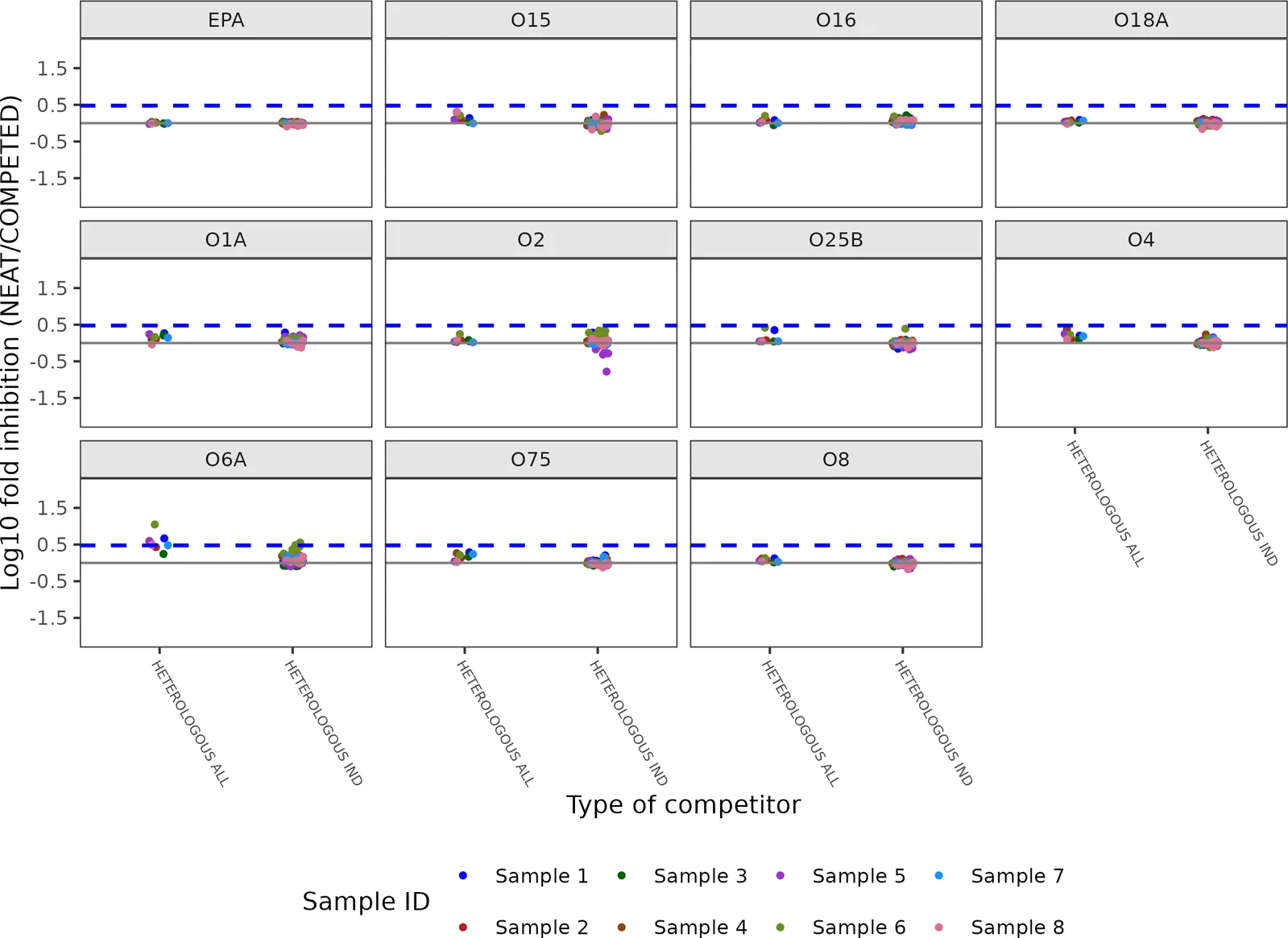
Evaluation of assay specificity with heterologous antigen competition. Log_10_ fold inhibition (neat/competed), Y-axis for heterologous competition. The colors indicate the sample ID. The gray horizontal solid line refers to a log_10_ fold inhibition equal to zero, and the blue horizontal dashed line refers to the log_10_ acceptance criteria for heterologous competition, i.e., log_10_ 3-fold inhibition.

The robustness evaluation further examined whether minor procedural variation affected assay performance.

### Robustness assessment

Assay robustness was validated by introducing variations in serum sample and detection antibody incubation times. A response surface design was employed, incorporating two continuous factors—serum incubation time and detection antibody incubation time—to estimate main effects, two-way interactions, and quadratic terms. The design included 10 experimental runs, each measured across 10 assay plates within a single assay run. On each plate, LQC and HQC samples were tested in nine replicates. For each run, the average log₁₀ concentration of LQC and HQC was calculated and modeled using a response surface approach, with all main effects, interactions, and quadratic terms included as fixed effects. Simulations are performed from the model to obtain the design space. The design space illustrates the proportion of future measurements outside the predefined acceptance criteria (also called the defect rate) across the experimental domain.

The assay demonstrated robustness across incubation times ranging from 90 to 150 min, with a maximum QC failure rate of 6%, which was well below the predefined 10% threshold. These results are summarized in Fig. 4 and 5.

**Fig 4:**
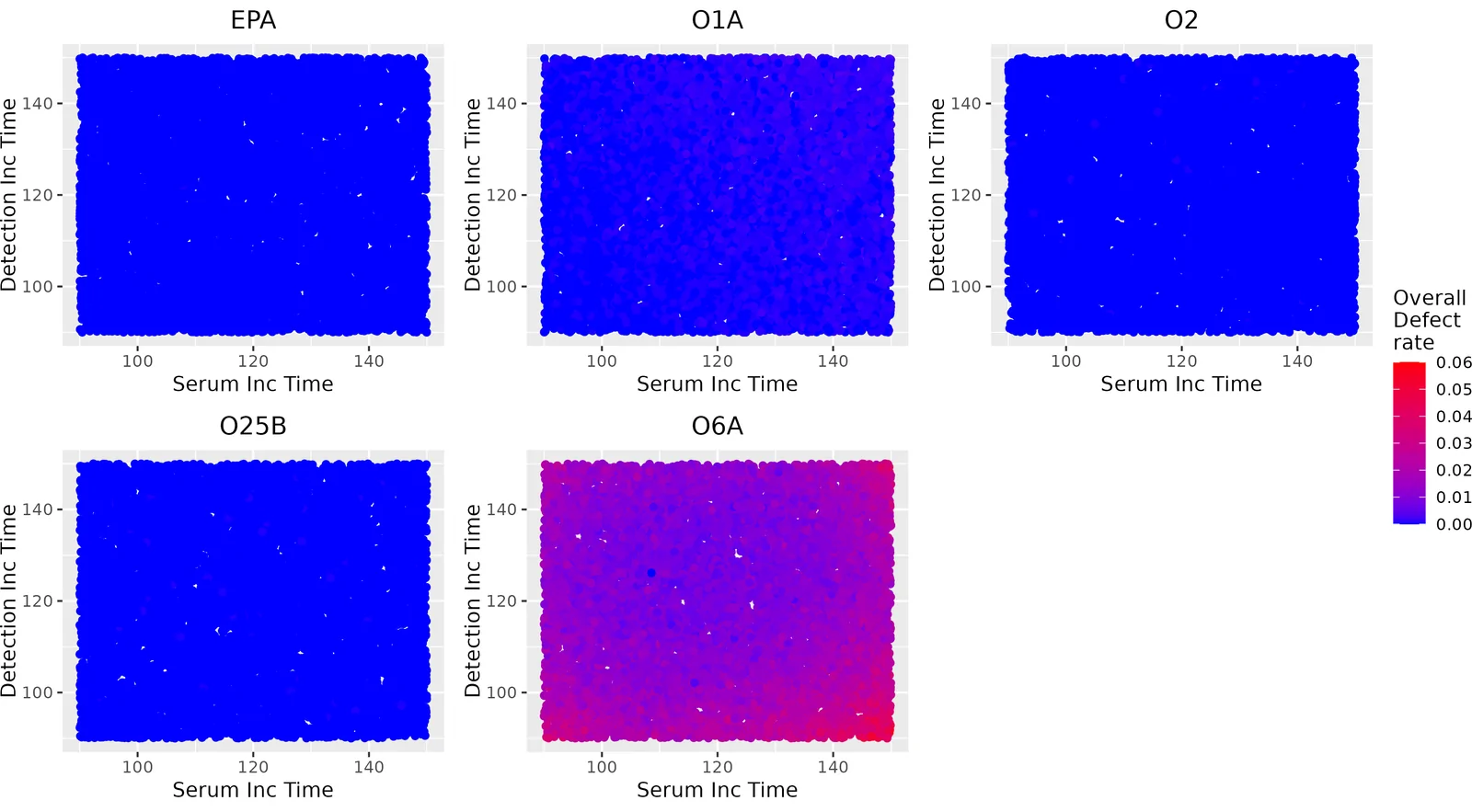
Assay robustness evaluation using design space across the complete experimental domain for the 4 O-antigens: O1A, O2, O25B, O6A. and carrier protein EPA. The blue to red color gradient indicates low to high joint probability to fall outside the acceptance criteria for at least one of the two QC samples, respectively. The legend provides an overview of the actual probabilities (overall defect rate) attached to the color gradient.

**Fig 5:**
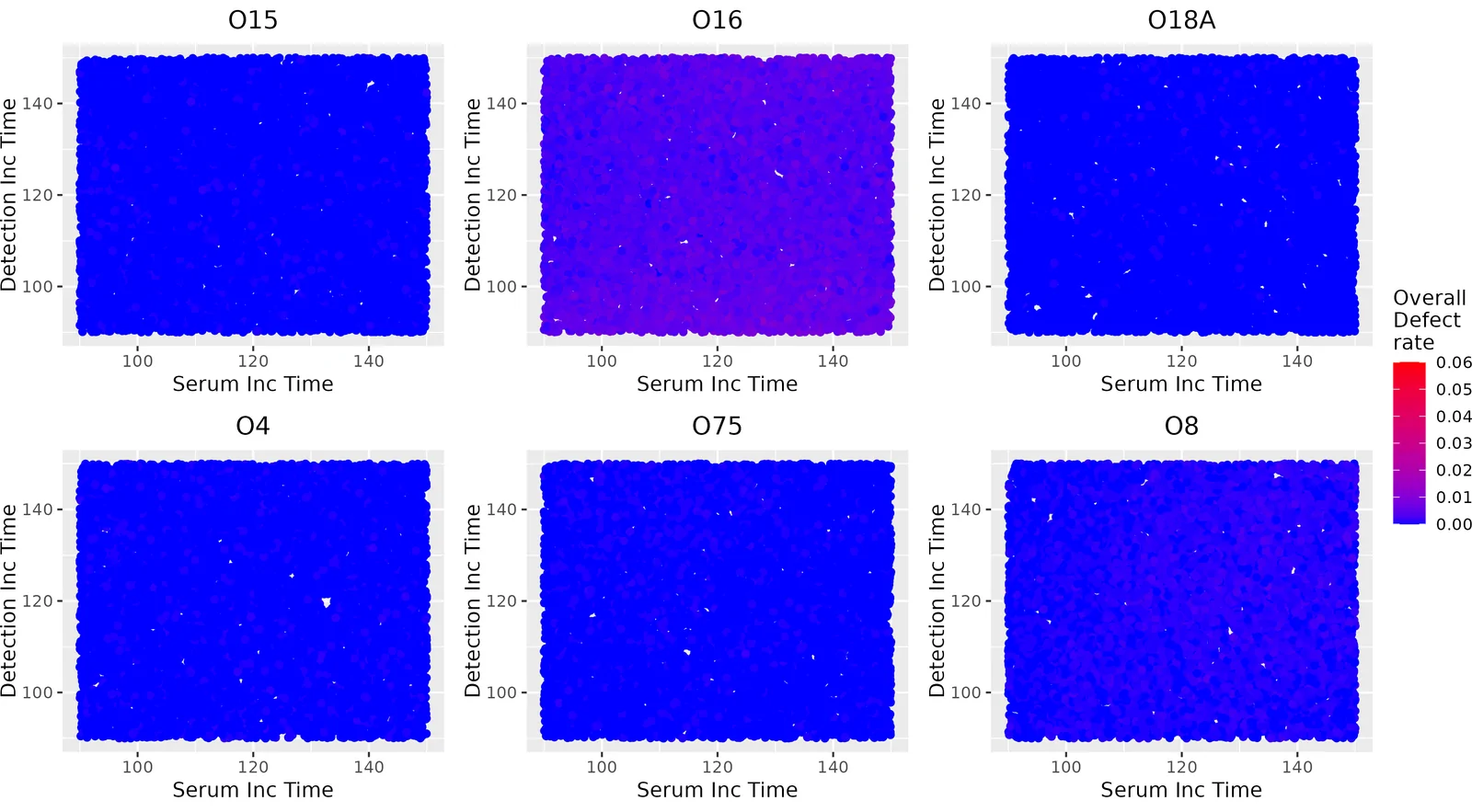
Assay robustness evaluation using design space across the complete experimental domain for the 6 O-antigens: O15, O16, O18A, O4, O75, and O8. The blue to red color gradient indicates low to high joint probability to fall outside the acceptance criteria for at least one of the two QC samples, respectively. The legend provides an overview of the actual probabilities (overall defect rate) attached to the color gradient.

In addition to analytical robustness, sample and plate stability were evaluated to support the operational use of the assay.

### Serum sample and plate stability

Pre-validation studies confirmed both the short-term and long-term stability of serum antibody titers, as well as the stability of pre-coated assay plates.

Across all evaluated storage conditions, the average log_₁₀_ differences between test and reference samples were minimal, with mean estimates close to zero. Dunnett-corrected 90% confidence intervals for these differences remained within predefined acceptance criteria across all serotypes and EPA (Fig. 6).

**Fig 6:**
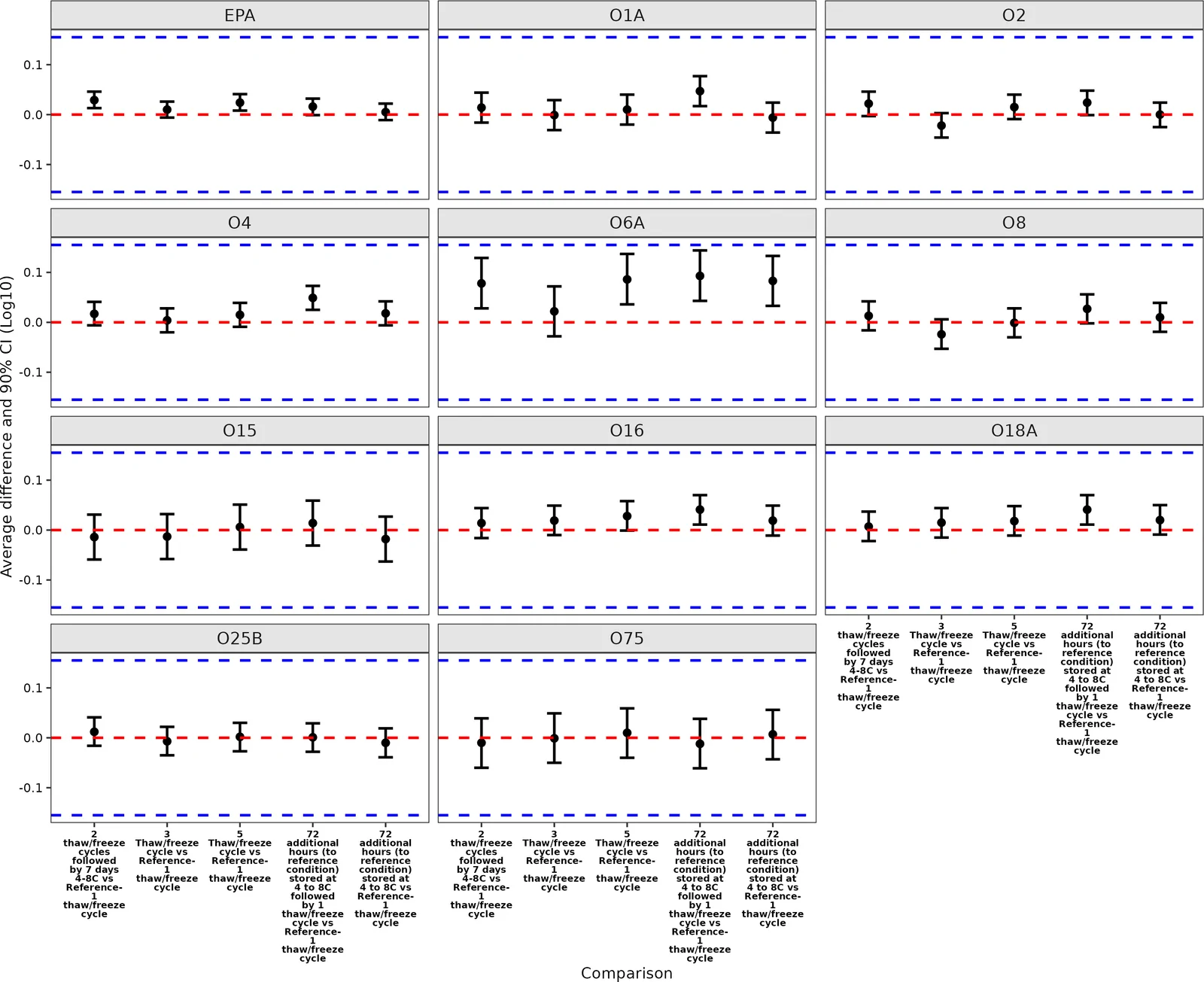
Estimated average difference and Dunnett-corrected 90% CI (log_10_) between test storage condition and reference storage condition for each serotype/EPA. The red dashed line represents a mean difference (log_10_) of 0, while the blue dashed lines represent the equivalence margin of (−0.155 log_10_, 0.155 log_10_).

Long-term stability was maintained over the evaluated period of up to 17 months. Mean log_₁₀_ differences between samples stored at 6, 12, and 17 months, and baseline (0 months) remained close to zero for all serotypes and EPA. Corresponding 90% confidence intervals were within predefined acceptance limits, confirming equivalence to baseline across all time points (Fig. 7).

**Fig 7:**
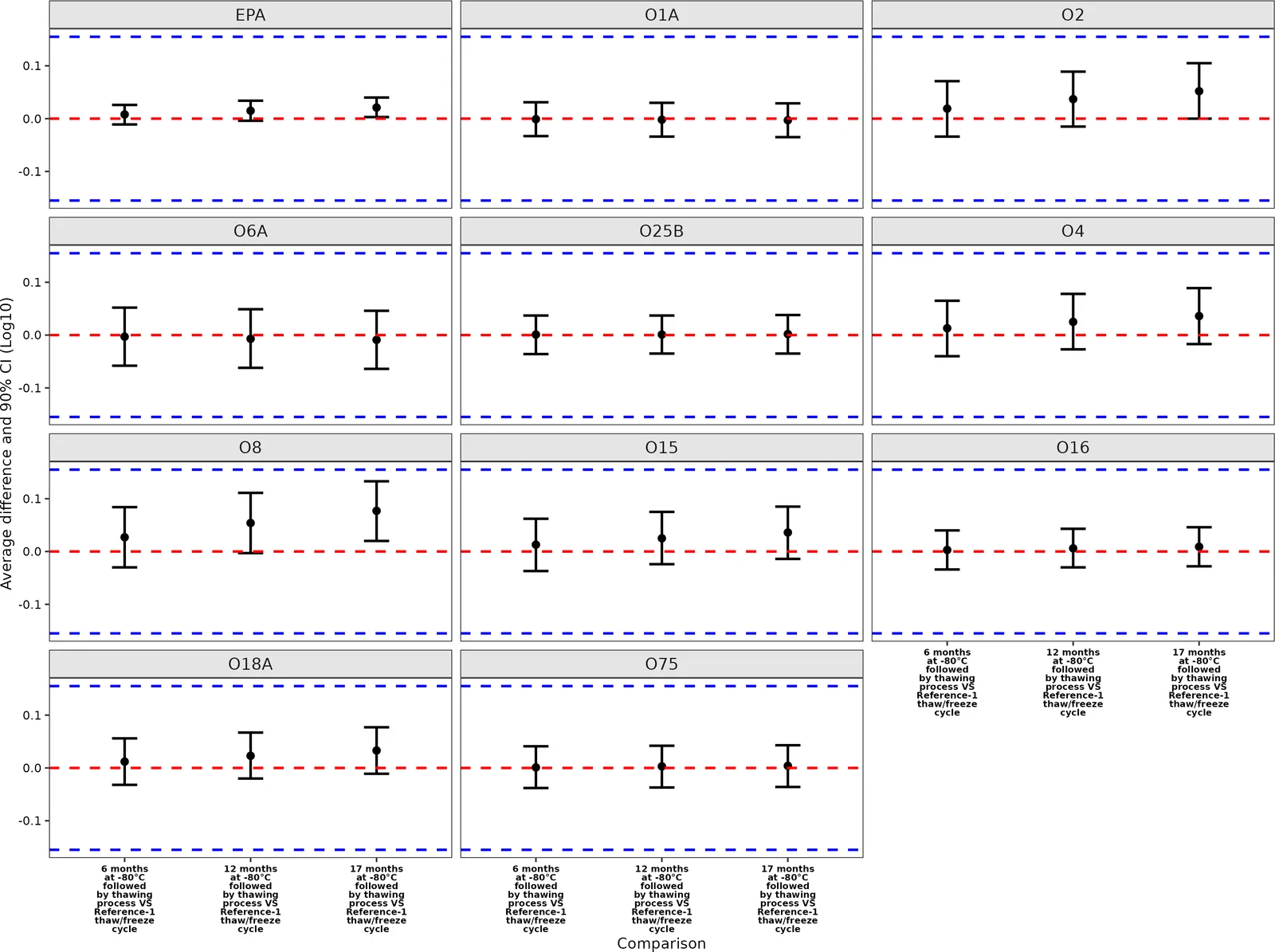
Estimated average difference and 90% CI (log_10_) of 0–17 months at −80°C followed by thawing process for each serotype/EPA. The red dashed line represents a mean difference (log_10_) of 0, while the blue dashed lines represent the equivalence margin of (−0.155 log_10_, 0.155 log_10_).

The stability of MSD assay plates was demonstrated over a period of up to 3 years when stored at 4°C. Systematic difference results are provided in Fig. 8 on the log_10_ scale. Acceptable systematic difference is observed between the test (T0) and reference (3 years) condition for all serotypes.

**Fig 8:**
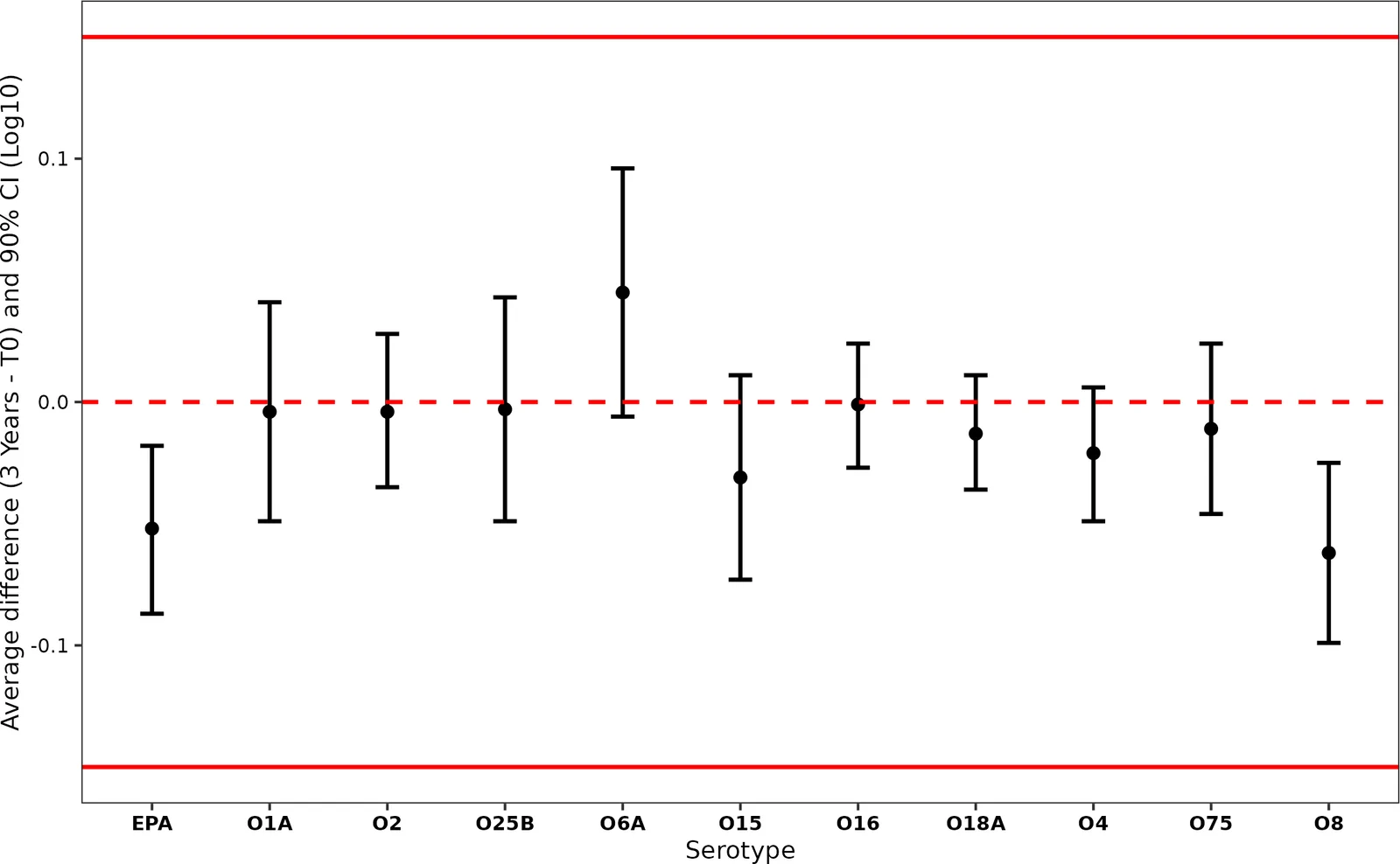
Systematic difference results of plate stability per serotype on the log_10_ scale. Average difference log_10_ concentration (3 years, T0) with 90% CI (Y-axis) versus serotype (X-axis). The red dashed line refers to a difference equal to zero. The red solid lines refer to the lower and upper acceptance criteria.

## DISCUSSION

This validation study confirms that the validated ECL immunoassay meets all critical analytical performance requirements for determining IgG antibody titers against multiple *E. coli* O-antigens and the EPA carrier protein in human serum. Assay ranges (LLOQ and ULOQ) for EPA, O15, O16, O18A, O1A, O2, O25B, O4, O75, and O8 were successfully established based on predefined criteria for linearity (90% CI slope between 0.80 and 1.25), precision (%CV for repeatability and intermediate precision ≤30%), and relative accuracy (%TE ≤50%) with minor limitations for O6A that did not impact overall suitability. The assay demonstrated consistent precision, with overall %CV IP below 16% for most antigens and acceptable linearity across the working range. Incurred sample testing yielded %CV (intermediate precision) values below 20%, supporting the reliability of the measurements. Acceptable dilutional linearity was observed at selected dilution factors, and QC sample performance confirmed the assay’s reliability and consistency across multiple runs. While O6A showed cross-reactivity in pooled heterologous competition experiments, all antigens met specificity acceptance criteria when heterologous antigens were tested individually. The O6A results were expected, and a similar trend was observed in early coating optimization studies on high-binding plates. Hydrophobic interactions can promote variable orientation and elevated surface density, increasing the presentation of conserved core epitopes and thereby enhancing apparent cross-reactivity. These effects can differ by O-antigen structure and chain length. Multiple plate options were evaluated during coating optimization, but high-binding plates provided the best overall performance (precision, linearity, and signal-to-noise) across the full antigen panel. Thus, high-binding plates were selected to maximize panel-level robustness despite a localized tradeoff for O6A that did not affect fitness for purpose for the assay. The assay also demonstrated robustness to variations in incubation times and maintained stability under defined storage conditions. Overall, the ECL immunoassay reliably determines antibody titers against multiple *E. coli* O-antigens and EPA in human serum, supporting its reliability for clinical use. Importantly, this validated ECL assay was accepted by regulatory authorities and implemented in the clinical evaluation of the ExPEC vaccine program (19, 20), including its use in the Phase III Embrace study. This global study evaluated the efficacy, safety, and immunogenicity of the ExPEC vaccine candidate in a large adult population, where the assay was used to measure antigen-specific antibody responses. Embrace trial was shut down in 2025 due to lack of efficacy (24). The successful application of this assay in a large-scale clinical trial further confirms its robustness and establishes the assay as a fit-for-purpose immunogenicity platform for clinical vaccine evaluation.
